# From Delivery to Design: Site-Specific Genome Engineering for Next-Generation CAR T-Cell Therapy

**DOI:** 10.3390/biomedicines14091925

**Published:** 2026-08-27

**Authors:** Hala Aldahshan, Abeer Al-Hubaysh, Dimah K. Alrabiah, Meshael Alturki, Reem R. Alkharji, Salwa Lin, Mohammed S. Aldughaim, Raef Albugami

**Affiliations:** 1 Department of Clinical Laboratory Sciences, College of Applied Medical Sciences, King Saud University, Riyadh 12372, Saudi Arabia; 2 Department of Biology, College of Science, Princess Nourah bint Abdulrahman University, Riyadh 11671, Saudi Arabia; aaalhubaysh@pnu.edu.sa; 3 Wellness and Preventive Medicine Institute, Health Sector, King Abdulaziz City for Science and Technology (KACST), Riyadh 11442, Saudi Arabia; dalrabiah@kacst.gov.sa (D.K.A.); mmsalturki@kacst.gov.sa (M.A.); 4 Research Department, Natural and Health Science Research Centre, Princess Nourah bint Abdulrahman University, Riyadh 11564, Saudi Arabia; rralkharji@pnu.edu.sa; 5 Department of Clinical Laboratory Sciences, Taif University, Taif 26521, Saudi Arabia; salwa.l@tu.edu.sa; 6 Ministry of Industry and Mineral Resources, King Fahd Street, Al-Muhammadiyah District, Riyadh 12363, Saudi Arabia; m.aldughaim@mim.gov.sa; 7 Pathology and Clinical Laboratory Administration, Riyadh Second Healthcare Cluster–KFMC, Riyadh 12231, Saudi Arabia

**Keywords:** CAR-T cell therapy, immune cell engineering, site-specific genome engineering, CRISPR/Cas9, targeted CAR integration, *TRAC* locus, homology-directed repair, HITI, non-viral manufacturing, genomic safe harbour

## Abstract

Chimeric antigen receptor (CAR) T-cell therapy has transformed the treatment of haematological malignancies, but conventional viral transduction results in semi-random genomic integration, contributing to heterogeneous CAR expression and potential insertional effects. Site-specific genome engineering offers an alternative by directing CAR insertion to defined genomic loci, allowing greater control over transgene expression and cellular function. This narrative review evaluates advances in site-specific CAR T-cell engineering, focusing on integration mechanisms, donor platforms, genomic loci, safety, and translational readiness. Homology-directed repair (HDR) and homology-independent targeted integration (HITI) are critically compared alongside viral and non-viral donor systems. Candidate loci are evaluated according to their functional consequences and level of evidence, with *TRAC* representing the most extensively characterised target, while *PDCD1*, *CD7*, *CD247*, and other loci offer distinct functional opportunities but remain supported by varying levels of preclinical and translational evidence. The review also examines genomic risks associated with targeted editing, including structural rearrangements and chromosome-scale abnormalities, and considers emerging applications in allogeneic, solid-tumour, and in vivo CAR T-cell engineering. Overall, site-specific integration provides a framework for linking CAR placement to therapeutic function, but no single locus or integration strategy is universally optimal. Clinical evidence remains limited relative to the expanding preclinical landscape, and genomic safety, scalable manufacturing, and clinical validation remain major priorities for translation.

## 1. Introduction

Chimeric antigen receptor (CAR) T-cell therapy has transformed the treatment of B-cell malignancies. Pivotal trials including ELIANA, JULIET, and ZUMA-3 established the clinical proof-of-concept for CD19-targeting CAR T-cell products across paediatric and adult populations with B-cell malignancies, reporting complete remission rates of 71–81% in relapsed or refractory B-cell acute lymphoblastic leukaemia [1,2], and durable responses, including complete remission in approximately 40% of patients, in relapsed or refractory diffuse large B-cell lymphoma [3]. Currently, all FDA-approved CAR T-cell products, including tisagenlecleucel (CTL019) and brexucabtagene autoleucel (KTE-X19), are generated by viral transduction using γ-retroviral or lentiviral vectors, with the potential for semi-random genomic integration of the CAR transgene [4,5].

While clinically effective, viral integration carries several well-recognised limitations. Semi-random genomic insertion produces positional effects, resulting in heterogeneous and often unstable CAR expression across individual T-cell clones [6]. This variability in transgene expression is influenced by the epigenetic landscape of the integration site; insertions within accessible euchromatin may support higher transcription, whereas integration near heterochromatin can promote reduced expression or gene silencing [7]. Compounding this positional variability is the lack of control over vector copy number (VCN) during transduction. Unregulated viral entry yields a broad, Poisson-like distribution of transgene copies per cell across the T-cell repertoire, resulting in variable signalling thresholds between individual clones [5]. Together, this polyclonal integration profile creates a highly heterogeneous product that compromises therapeutic consistency. In vivo, hyper-expressing clones often undergo chronic, antigen-independent tonic stimulation, leading to accelerated activation-induced cell death (AICD) and premature functional exhaustion [8,9,10]. In contrast, hypo-expressing clones fail to cross the activation threshold required for robust tumour lysis [11,12]. Beyond these single-cell expression dynamics, integration at or near proto-oncogenes or active transcriptional units raises a clinically documented risk of insertional mutagenesis, as demonstrated in early γ-retroviral gene therapy trials in which vector integration near the *LMO2* locus resulted in clonal T-cell expansion and leukaemogenesis [13,14]. This genotoxicity is primarily driven by the enhancer and promoter activity of viral long terminal repeats (LTRs), which can aberrantly activate neighbouring genes over considerable genomic distances [15,16]. Accordingly, vector design and genomic integration site selection have emerged as critical determinants of safety in engineered T-cell therapies. Lentiviral vectors exhibit an improved genotoxicity profile compared with γ-retroviral systems; however, they are not entirely devoid of insertional effects, as integration events can still influence clonal dynamics depending on genomic context and vector design [5,16]. Polyclonal integration further contributes to product heterogeneity, resulting in variable CAR expression levels and signalling potency across the T-cell repertoire [6,17].

Beyond safety and product consistency, autologous viral CAR T-cell manufacturing remains laborious and costly. List prices for currently FDA-approved CAR T-cell products range from $373,000 to $475,000 per infusion, with drug acquisition representing approximately 74% of the total per-patient cost. Real-world total costs, including lymphodepletion, hospitalisation, toxicity management, and post-treatment care, consistently exceed $700,000 and surpass $1,000,000 in a substantial proportion of cases [18,19]. Manufacturing timelines typically span 2–4 weeks from leukapheresis to infusion [20].

**Figure 1:**
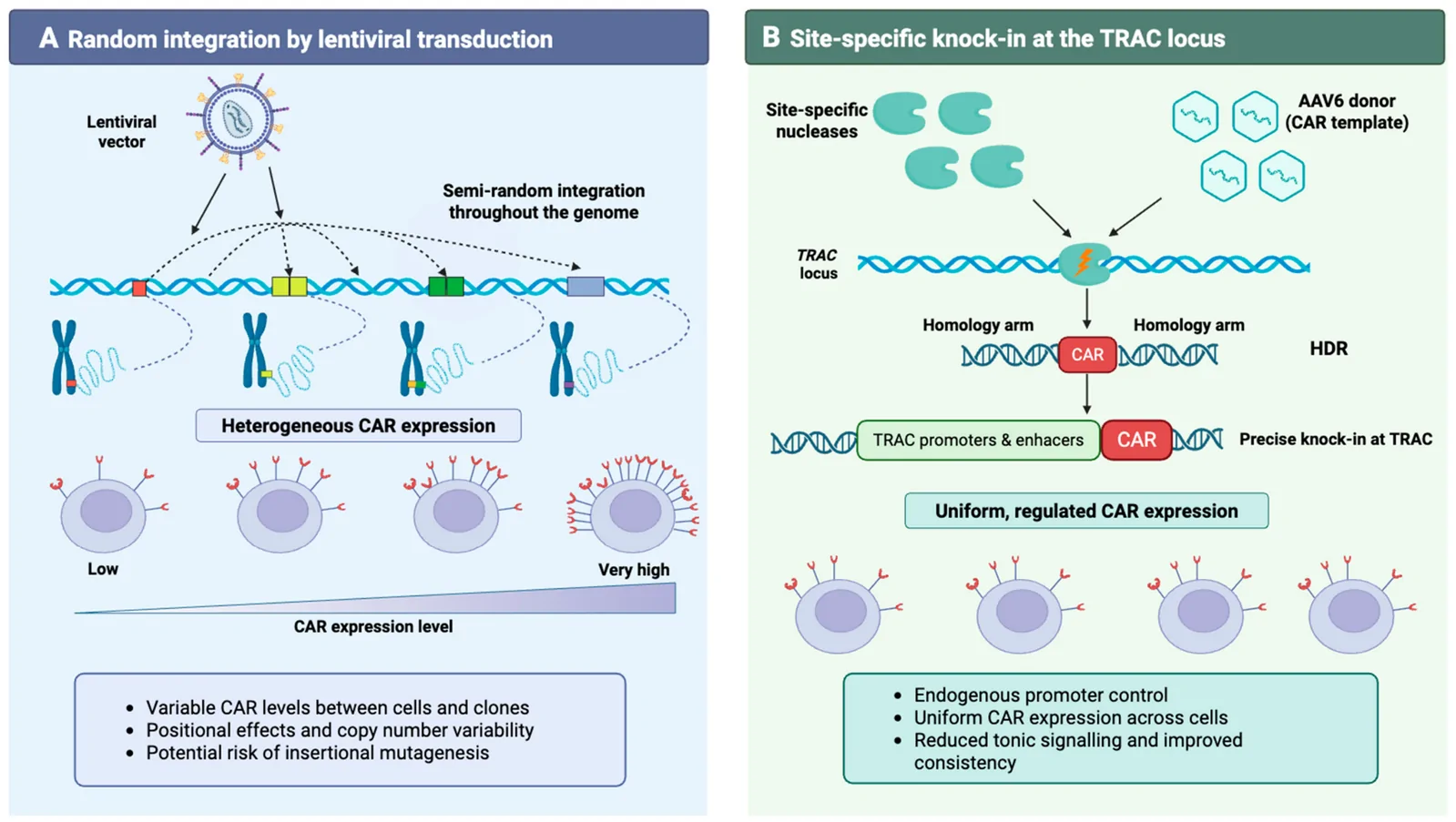
Transition from semi-random viral integration to programmable site-specific CAR-T genome engineering. (**A**) Conventional viral transduction results in semi-random genomic integration of the CAR transgene, leading to heterogeneous expression, positional effects, and variable functional responses across T-cell populations. (**B**) Site-specific genome editing enables targeted CAR insertion (e.g., at the *TRAC* locus), placing the transgene under endogenous regulatory control and promoting more uniform CAR expression and reduced tonic signalling. Created in BioRender. Ruiz, M. (2026) https://BioRender.com/wpssrzy, accessed on 23 August 2026.

These limitations have driven the development of site-specific genome engineering approaches that insert the CAR transgene at defined genomic loci under endogenous regulatory control, resulting in more uniform and physiologically regulated CAR expression (Figure 1) [8]. Eyquem et al. (2017) demonstrated that targeting the CAR to the *TRAC* locus via CRISPR/Cas9-mediated homology-directed repair (HDR) results in uniform surface expression, reduced tonic CAR signalling, delayed T-cell exhaustion, and superior antitumour activity in preclinical models [8,21]. Subsequent studies have replicated and extended these findings across additional genome editing platforms and experimental systems, including targeted CAR integration into the *TRAC* locus using alternative nucleases and donor templates, supporting the robustness and generalisability of site-specific CAR engineering strategies [22,23,24].

Key messages:

- Semi-random viral integration can generate heterogeneous CAR expression and positional effects.
- Site-specific *TRAC* knock-in enables endogenous regulation and more uniform CAR expression.
- Integration site and expression architecture can influence CAR-T functional consistency.

Two principal strategies define established site-specific CAR integration: homology-directed repair (HDR), which uses a donor DNA template to achieve precise transgene insertion, and homology-independent targeted integration (HITI), which exploits the non-homologous end-joining (NHEJ) pathway for cell-cycle-independent knock-in [8,25,26]. Integration mechanism, donor format, genomic locus, and expression architecture therefore represent interconnected determinants of site-specific CAR design.

Accordingly, this review focuses specifically on site-specific genomic integration of CAR transgenes, rather than CAR T-cell generation or genome editing more broadly. It examines how integration mechanism, donor format, genomic locus, and CAR expression architecture influence CAR T-cell function, genomic safety, manufacturing feasibility, and clinical translation. Following a brief description of the literature search strategy, Section 2 reviews the major genome editing strategies for site-specific CAR integration, including programmable nuclease platforms, HDR, HITI, and emerging DSB-independent integration approaches, together with their comparative advantages and limitations. Section 3 evaluates donor template and delivery platforms and the genomic safety considerations associated with targeted integration. Section 4 examines candidate genomic loci according to their biological rationale, functional consequences, and level of translational evidence, while distinguishing true site-specific CAR knock-in from *TRAC* disruption combined with separate CAR delivery. Section 5 considers emerging CAR T-cell engineering paradigms and applications, including autologous versus allogeneic engineering, in vivo CAR T-cell generation, and applications in solid tumours. Section 6 evaluates clinical translation and manufacturing readiness across the major site-specific integration strategies. Together, these comparisons define the current evidence base, limitations, and translational priorities for site-specific CAR T-cell engineering.

### Literature Search Strategy

This narrative review is based on searches of PubMed, MEDLINE, Web of Science, and ClinicalTrials.gov covering January 2012 to August 2026, supplemented by targeted forward citation tracking of key primary studies. Particular attention was given to recent studies relevant to site-specific CAR T-cell engineering, including work on in vivo CAR generation, intron-targeted knock-in, CD3ζ-targeted integration, and enveloped delivery vehicle platforms. Search terms included “CAR T CRISPR knock-in”, “site-specific CAR insertion”, “*TRAC* CAR T”, “non-viral CAR T cell”, “homology-directed repair T-cell”, “homology-independent targeted integration”, “HITI CAR T”, “allogeneic CAR T clinical trial”, “in vivo CAR T delivery”, “armoured CAR T”, “nanoplasmid T-cell engineering”, “rAAV6 CAR knock-in”, “PDCD1 PD-1 CAR”, “CD3ζ CD247 CAR knock-in”, “extragenic safe harbour T-cell”, and “in vivo genome editing T-cell”. The search was restricted to English-language publications in peer-reviewed journals. Conference abstracts and company communications were cited only where peer-reviewed data were unavailable and were explicitly identified as such. Reference lists of included primary studies were manually screened for additional relevant sources. As this is a narrative review, PRISMA reporting was not applied.

## 2. Genome Editing Strategies for Site-Specific CAR Integration

Site-specific CAR T-cell engineering commonly relies on targeted nuclease-induced DNA double-strand breaks (DSBs), which enable CAR transgene insertion via two distinct repair pathways: HDR, using an exogenous donor template for precise knock-in [8], and HITI, which exploits the NHEJ pathway for cell-cycle-independent insertion [25]. These two strategies, together with the donor platforms that support them, are the primary focus of this section.

Recent developments have expanded the ability to control transgene integration site, expression architecture, and genomic context, increasing interest in site-specific CAR engineering approaches. Understanding how integration mechanism, donor platform, and genomic locus interact to shape T-cell function will be critical for the next generation of safer, more effective, and scalable cellular immunotherapies.

### 2.1. Programmable Genome Editing Platforms

All nuclease-based approaches rely on the induction of a DSB at a defined genomic locus, which is repaired by the cell through one of two pathways: the error-prone non-homologous end joining (NHEJ) pathway, which generates insertions or deletions (indels) and gene disruption, or the homology-directed repair (HDR) pathway, which enables precise transgene integration using an exogenous donor template. These repair pathways form the mechanistic basis of current genome engineering strategies [27,28].

Before the widespread adoption of CRISPR/Cas9, zinc-finger nucleases (ZFNs) had already demonstrated site-specific transgene integration in primary human T-cells. ZFN mRNA combined with AAV6 donor delivery achieved homology-directed repair (HDR)-mediated transgene knock-in at the *CCR5* and *AAVS1* loci, with efficiencies exceeding 40% in primary CD4+ and CD8+ T-cells [29].

Subsequently, CRISPR/Cas9 emerged as the dominant platform for site-specific CAR T-cell engineering because RNA-guided targeting is simpler to reprogram than the protein engineering required for each new nuclease target and readily supports multiplex editing [30,31]. CRISPR/Cas9 is typically delivered to T-cells as a ribonucleoprotein (RNP) complex comprising Cas9 protein and a single guide RNA (sgRNA), usually by electroporation. RNP delivery is generally preferred over plasmid-based expression because transient intracellular exposure limits off-target activity, reduces immunogenicity, and eliminates the risk of unintended Cas9 sequence integration [32].

### 2.2. HDR-Mediated CAR Knock-In

**Figure 2:**
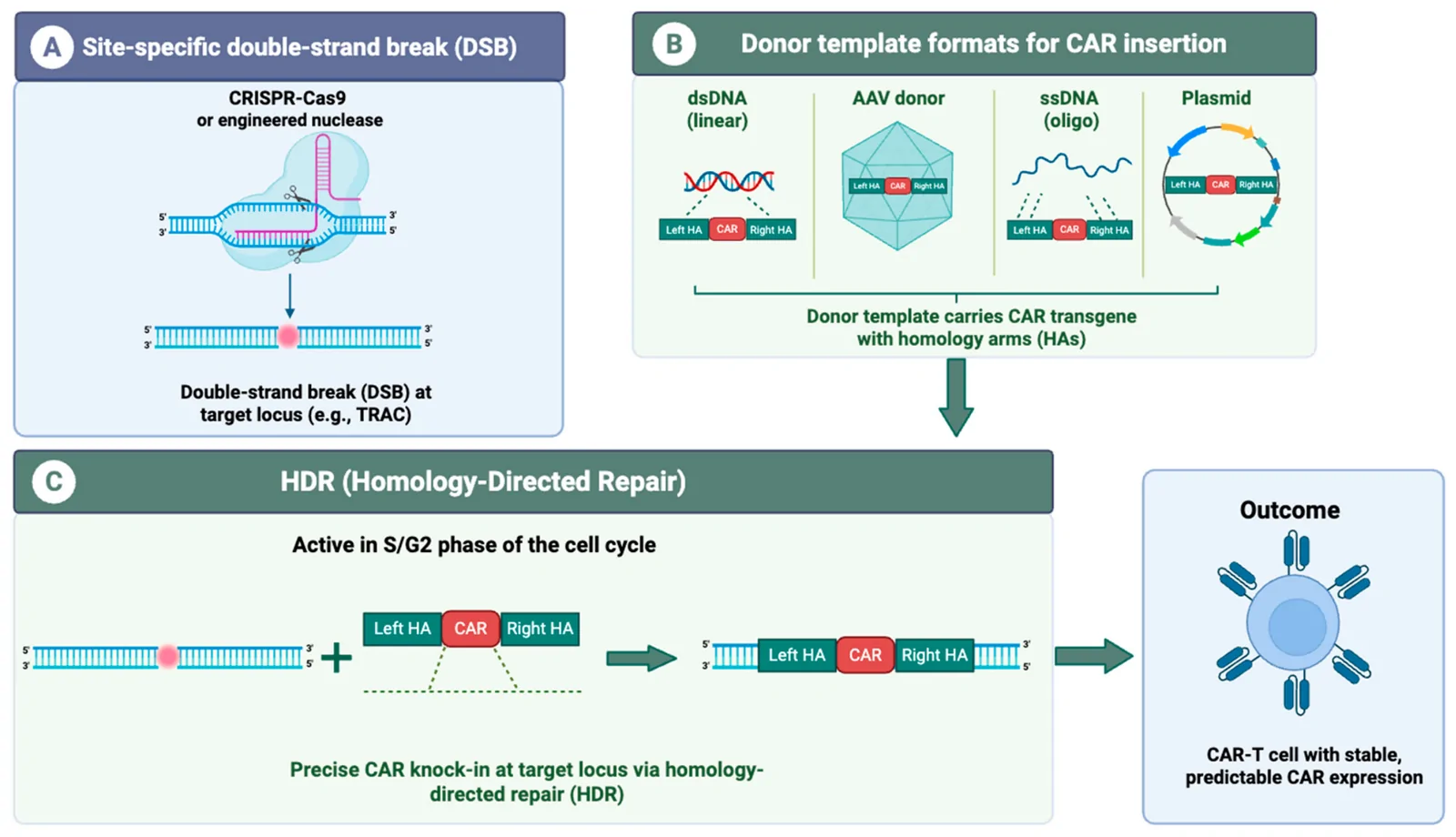
Homology-directed repair enables precise CAR transgene knock-in. (**A**) Site-specific nucleases, such as CRISPR-Cas9, introduce a double-strand break (DSB) at a defined genomic locus (e.g., *TRAC*). (**B**) Donor templates carrying the CAR transgene flanked by homology arms can be delivered in multiple formats, including linear double-stranded DNA (dsDNA), adeno-associated virus (AAV) vectors, single-stranded DNA (ssDNA), or plasmid DNA. (**C**) Homology-directed repair (HDR), which is most active during the S/G2 phases of the cell cycle, uses these homology arms to direct precise CAR insertion at the target locus. Created in BioRender. Ruiz, M. (2026) https://BioRender.com/wpssrzy, accessed on 23 August 2026.

Homology-directed repair (HDR) remains the dominant strategy for precise site-specific CAR transgene integration in primary T-cells, as demonstrated by multiple studies targeting the *TRAC* locus using diverse nuclease platforms and donor delivery systems [8,22]. HDR uses an exogenous donor DNA template flanked by homology arms corresponding to genomic sequences adjacent to the double-strand break, enabling precise transgene integration at the cleavage site (Figure 2) [11,33]. HDR activity is largely restricted to the S and G2 phases of the cell cycle, when homologous recombination is favoured. Accordingly, T-cell activation and cell-cycle status can substantially influence HDR efficiency, and many established editing protocols use ex vivo stimulation to promote proliferation and enhance targeted integration [34,35]. Site-specific knock-in of a CD19-specific CAR into the *TRAC* locus using CRISPR/Cas9-mediated HDR and an rAAV6 donor template achieved frequencies exceeding 40% at the highest AAV6 multiplicity of infection tested (1 × 10^6^), alongside a *TRAC* knockout rate of approximately 70% [8]. In addition to *TRAC*, targeted CAR integration has been reported at alternative loci including *PDCD1*, *CD7*, AAVS1, and *CCR5*, each selected for a distinct functional or regulatory rationale, as reviewed in Section 4 [23,29,36,37,38].

Despite its precision, HDR has two important biological limitations for CAR T-cell engineering. First, HDR competes with non-homologous end joining (NHEJ) at nuclease-induced DSBs. Cells that repair the target site through NHEJ rather than HDR remain donor-negative, reducing the yield of correctly integrated CAR T-cells. Transient inhibition of DNA-dependent protein kinase catalytic subunit (DNA-PKcs) using compounds such as **M3814** can suppress NHEJ and increase HDR-mediated integration [39,40], although the effects of manipulating DNA repair pathways on long-term T-cell fitness require further investigation.

Second, the cell-cycle dependence of HDR may influence the cellular composition of the edited product. Naïve and stem cell memory T-cells possess favourable persistence properties [41], but their relatively quiescent state may limit HDR efficiency compared with more actively proliferating T-cell populations. Whether this results in clinically meaningful changes in the phenotype or persistence of the final CAR T-cell product remains unclear.

Additional limitations related to donor format, including cargo capacity, template-associated cytotoxicity, manufacturing complexity, and unintended donor integration, are discussed separately in Section 3.

Key messages:

- Targeted nucleases create a defined genomic DSB for CAR insertion.
- Multiple donor formats can provide the homology-directed CAR template.
- HDR enables precise, site-specific CAR integration at the selected locus.

### 2.3. NHEJ-Mediated CAR Knock-In: Homology-Independent Targeted Integration (HITI)

Homology-independent targeted integration (HITI) exploits the non-homologous end-joining (NHEJ) pathway for site-directed transgene insertion and, critically, operates throughout the cell cycle, including quiescent G0/G1 cells [25]. Unlike HDR, HITI does not depend on the S/G2-associated homologous recombination machinery and can therefore support targeted integration in non-dividing cells [25]. This feature may permit editing of less-activated T-cell populations, although whether this translates into improved preservation of naïve or stem cell memory phenotypes and greater in vivo persistence remains to be established [26].

Mechanistically, the HITI donor construct is flanked by engineered Cas9 recognition sequences oriented as reverse complements of the genomic target. Cas9 cleavage therefore occurs both at the genomic locus and at the donor plasmid, releasing a linear transgene cassette that can be inserted into the genomic break through NHEJ-mediated repair [25]. Orientation control is achieved through a re-cleavage mechanism. Reverse-orientation insertions recreate an intact Cas9 recognition sequence and remain susceptible to further Cas9 cleavage, whereas correctly oriented insertions disrupt the target motif and are preferentially retained [25]. In the reported CAR T-cell implementation, junction sequencing identified NHEJ-associated indels, including small insertions and deletions at the integration junctions [26].

HITI-mediated CAR knock-in has been demonstrated in primary human T-cells using a nanoplasmid donor to integrate an anti-GD2 CAR at the *TRAC* locus [26]. In a direct head-to-head comparison within the same experimental system, HITI yielded at least twofold more CAR-positive cells than nanoplasmid-based HDR, potentially reflecting its cell-cycle independence and differences in donor-associated cytotoxicity [26]. To achieve clinical-grade purity, CRISPR EnrichMENT (CEMENT) was developed as a pharmacological selection strategy in which a mutant dihydrofolate reductase cassette is co-integrated alongside the CAR transgene, conferring methotrexate resistance to correctly edited cells and enabling their selective expansion [26]. Following CEMENT, a 14-day fully non-viral manufacturing process generated 5.5 × 10^8^ to 3.6 × 10^9^ GD2-CAR-positive cells at approximately 80% purity from a starting population of 5 × 10^8^ T-cells [26].

Functionally, HITI/CEMENT products demonstrated cytolytic potency and antitumour efficacy comparable to retroviral CAR-T controls in a GD2-positive xenograft model [26]. However, these findings should be interpreted cautiously because the comparator involved randomly integrated, exogenous promoter-driven CAR expression rather than *TRAC*-targeted HDR, and evaluation was limited to a single tumour model. Direct comparative evidence regarding long-term persistence, exhaustion, and performance across different tumour microenvironments therefore remains lacking. CEMENT also introduces an additional pharmacological enrichment step, which adds complexity to downstream manufacturing and product characterisation.

Homology-mediated end joining (HMEJ) represents an emerging alternative that seeks to combine efficient non-viral targeted integration using short regions of sequence homology. Targeted integration of 1–6.7 kb payloads at multiple loci in primary human T-cells has been reported with efficiencies of up to 70% and post-editing viability exceeding 80% [42]. HMEJ uses short (~48 bp) homology arms flanked by synthetic Cas9 recognition sequences, enabling donor linearisation and targeted integration during a favourable post-activation repair window [42]. In a matched comparison, HMEJ achieved 52.4% integration compared with 42.8% using conventional HDR and showed reduced sensitivity to donor-target sequence mismatches [42]. Engineered cells maintained proliferative capacity, low exhaustion-marker expression, and antitumor activity in vitro and in vivo [42].

HMEJ achieved high non-viral knock-in efficiency with precise targeted integration in the reported experimental system [42]. However, these findings remain preclinical and have not established superiority across different CAR constructs, genomic loci, or long-term in vivo settings. Thus, whether HMEJ offers a reproducible advantage over established integration strategies remains uncertain, particularly with respect to genomic safety, long-term persistence, and clinical scalability.

### 2.4. Comparative Summary: HDR Versus NHEJ/HITI

No single integration strategy currently performs optimally across all relevant parameters. HDR remains the most extensively characterised approach for precise targeted integration and can support endogenous CAR regulation when appropriately designed, whereas HITI provides cell-cycle-independent, fully non-viral engineering at the expense of less predictable junction architecture. HMEJ may offer an intermediate balance between knock-in efficiency and junction precision, although its evidence base remains predominantly preclinical [42]. Accordingly, Table 1 compares HDR, HITI, and HMEJ across knock-in efficiency, post-editing viability, junction fidelity, genomic safety considerations, CAR expression architecture, cargo flexibility, and functional performance.

Key messages:

- HDR provides high junction precision and supports endogenous CAR regulation but is cell-cycle dependent and donor-format sensitive.
- HITI enables cell-cycle-independent KI but produces less predictable NHEJ-mediated junctions.
- HMEJ combines promising KI efficiency with improved junction precision, although evidence remains predominantly preclinical.

### 2.5. Emerging DSB-Independent Integration Strategies

The genomic risks associated with nuclease-induced double-strand breaks (DSBs) have stimulated interest in alternative strategies for site-specific integration that reduce or avoid reliance on DSB-mediated repair. Prime editing and Bxb1 serine integrase-based systems represent two emerging approaches with potential relevance to site-specific CAR T-cell engineering.

Prime editing uses a Cas9 nickase fused to an engineered reverse transcriptase together with a prime-editing guide RNA (pegRNA), enabling programmable sequence modification without generating a conventional DSB [46]. Although conventional prime editing is constrained in the size of sequences that can be inserted, twin prime editing (TwinPE) extends this principle to larger DNA integration by installing recombinase-compatible sequences at defined genomic sites [47]. More recently, PRIME-In demonstrated non-viral, DSB-independent targeted integration of large DNA cargos in primary human T-cells, including integration of an approximately 3 kb CAR cassette at efficiencies of up to 50% [48]. This provides direct evidence that prime-editing-based approaches can support therapeutically relevant CAR-sized integration without conventional nuclease-induced DSBs.

Bxb1 serine integrase provides a complementary strategy for targeted large-cargo integration. Bxb1 catalyses unidirectional site-specific recombination between defined attB and attP attachment sites and can therefore mediate integration without relying on cellular HDR [49]. However, conventional Bxb1-based approaches require compatible attachment sites to be established at the genomic target, limiting direct application to endogenous loci. Prime-assisted site-specific integrase gene editing (PASSIGE) combines prime editing with Bxb1-mediated integration to establish programmable landing sites and subsequently insert larger DNA cargos [50]. Evolved Bxb1 variants have further increased targeted integration efficiency, including at therapeutically relevant genomic loci, supporting the potential of prime editing–integrase combinations for programmable large-cargo insertion [50].

## 3. Donor Template and Delivery Platforms: From Viral to Non-Viral Engineering

The donor template is a major determinant of editing efficiency, cellular viability, cargo capacity, and translational feasibility in site-specific CAR T-cell engineering. Although programmable nucleases create the genomic entry point for transgene insertion, successful knock-in also depends on the format used to deliver the donor template. Viral and non-viral donors differ in cellular uptake, innate immune sensing, payload capacity, scalability, and compatibility with different integration mechanisms. This section compares the principal donor platforms used for site-specific CAR knock-in, including rAAV6, linear dsDNA, conventional plasmids, nanoplasmids, and long ssDNA-based formats. A mechanistically distinct Cas9-containing lentiviral virus-like particle (Cas9-VLP) platform is discussed separately because it combines site-specific gene disruption with semi-random CAR integration rather than true site-specific CAR knock-in.

### 3.1. rAAV6

rAAV6 is a highly efficient HDR donor for primary human T-cells: CRISPR/Cas9 editing with an AAV6 repair template achieves site-specific knock-in at *TRAC* exceeding 40% at a multiplicity of infection of 10^6^, against a background *TRAC*-knockout frequency of ~70% [8]. Sialic acid is the predominant attachment factor, with N-linked α2,3 and α2,6 sialic acid residues serving as the primary binding determinants, while heparan sulfate proteoglycan interactions, though detectable, play only a secondary role in productive cellular entry [51,52].

Because rAAV6 delivers its cargo as single-stranded DNA, it does not activate the cGAS-STING innate sensing pathway as strongly as exogenous double-stranded DNA donors, which is one reason it preserves T-cell viability relative to linear dsDNA or plasmid formats at equivalent editing doses [45,53]. rAAV6 remains one of the most extensively characterised HDR donor platforms for site-specific CAR integration. Using AAV6 repair matrices, targeted integration of CAR constructs in primary human T-cells has been demonstrated at *TRAC* [8,22] and *B2M* [8], and at *PDCD1* and *IL2RA* [54].

Several limitations accompany rAAV6 donor delivery. GMP-grade vector production is technically demanding and adds time and cost to manufacturing [44,45]. The ~4.7 kb packaging capacity also restricts practical insert size once homology arms and other required sequences are included. Persistent unintegrated AAV genomes may additionally undergo unintended capture at nuclease-induced DSBs, making donor-integration profiling an important component of genomic safety assessment [45]. These limitations have contributed to the development of non-viral donor formats with greater cargo flexibility and simpler manufacturing requirements.

Engineered AAV variants are also being investigated for T-cell targeting. Ark313, evolved from the AAV6 capsid, achieves efficient transduction of murine T-cells [55]. AAV6-M2 enables in vivo CAR expression in human T-cells following systemic administration, converting up to 77.5% of CD8^+^ T-cells into CAR T-cells in humanised immune-system mouse models [56]. These variants primarily address cellular targeting and in vivo delivery rather than the intrinsic limitations of rAAV6 as an ex vivo HDR donor platform.

### 3.2. Linear dsDNA

Linear dsDNA represents one of the simplest non-viral donor formats and has been used for targeted TCR replacement at the *TRAC* locus in primary human T-cells [11]. Its principal limitation is donor-associated cytotoxicity. Electroporated dsDNA activates cytosolic DNA-sensing pathways, including cGAS-STING, leading to inflammatory signalling and reduced T-cell viability [53]. This effect is influenced by donor concentration and delivery conditions and can reduce the recovery of successfully edited cells. Modification of electroporation conditions has been shown to attenuate dsDNA-associated innate sensing and improve viable T-cell recovery [53].

Unmodified linear dsDNA generally achieves lower knock-in efficiency than optimised donor formats, although reported efficiencies vary according to donor size, target locus, dose, and experimental conditions [11,40]. Addition of truncated Cas9 target sequences (tCTS) to the donor ends can substantially improve HDR efficiency by promoting Cas9 RNP binding and donor nuclear co-localisation, with approximately two- to fourfold increases reported in primary cells [40]. Accordingly, comparisons of linear dsDNA with other non-viral donor platforms should distinguish between unmodified and tCTS-modified templates.

### 3.3. Conventional Circular Plasmids

Conventional circular plasmids provide a relatively inexpensive and scalable non-viral donor format but contain substantial prokaryotic backbone sequences, including antibiotic-resistance genes, bacterial origins of replication, and unmethylated CpG motifs [57]. These non-therapeutic DNA sequences increase the amount of exogenous DNA introduced during electroporation and may contribute to innate immune activation and cytotoxicity [53,58].

Incorporating Cas9-cleavage sequences (CCS) into the plasmid can improve HDR by enabling Cas9-mediated donor linearisation and release of the HDR template. In primary human T-cells, CCS-modified size-reduced plasmids improved CAR knock-in at the *TRAC* locus relative to unmodified circular plasmids [59]. Cas9-CLIPT extends this strategy using a Cas9-cleavable nanoplasmid that is linearised in vitro before electroporation. The approach achieved up to 60% knock-in under NHEJ inhibition for 2–6 kb transgenes, including a 5.0 kb GD2-CAR at *TRAC*, and yielded a product enriched for a stem cell memory phenotype [60].

These approaches demonstrate that plasmid performance depends not only on backbone composition and size but also on donor configuration and the method used to generate the linear HDR substrate.

### 3.4. Nanoplasmids

Nanoplasmids reduce several limitations associated with conventional plasmid donors by eliminating antibiotic-resistance genes and substantially reducing CpG-rich prokaryotic backbone sequences, while retaining the practical advantages of circular DNA [57]. The nanoplasmid backbone is approximately 430 bp, substantially smaller than the 2–4 kb backbones of conventional plasmids, and uses an antibiotic-free R6K-dependent selection system [57].

Nanoplasmid donors have been applied to both HDR- and HITI-mediated targeted integration [26,58]. In HITI-based CAR engineering, nanoplasmid donors achieved 14–35% unselected CAR^+^ T-cell frequencies at clinical manufacturing scale and outperformed linear dsDNA and conventional plasmid donors at equivalent doses [26]. For HDR-mediated knock-in, a matched, dose-titrated comparison of linear dsDNA, conventional plasmid, and nanoplasmid donors at the *TRAC* locus in primary human CD8^+^ T-cells showed clear format-dependent differences [58]. Linear dsDNA achieved 28.8–32.1% knock-in but reduced cell recovery, whereas conventional plasmid reached 33.5–44% only at doses that compromised viability. Nanoplasmid donors achieved 36.2–46.6% knock-in while preserving viability and expansion, resulting in approximately twofold and three-fold greater edited-cell yields than conventional plasmid and linear dsDNA donors, respectively [58]. Nanoplasmid-mediated integration of engineered TCRs and a CD19-specific CAR at *TRAC* also generated cells that retained antigen-specific cytokine production and cytolytic activity [58].

Nanoplasmids and CCS-modified size-reduced plasmids should therefore be distinguished: the former primarily minimise prokaryotic backbone content, whereas the latter additionally incorporate Cas9-cleavage sequences to facilitate generation of a linear HDR substrate. Minicircle vectors similarly reduce prokaryotic backbone sequences through site-specific recombination; however, their additional production steps and lower manufacturing yields may complicate scalability [61].

### 3.5. Long ssDNA and Hybrid ssCTS Donor Templates

Long single-stranded DNA (ssDNA) provides a non-viral donor format that can reduce donor-associated cytotoxicity while supporting targeted integration of therapeutic transgenes. At the multi-kilobase sizes required for CAR constructs, long ssDNA donors engage canonical RAD51-dependent homology-directed repair (HDR), rather than the RAD51-independent single-strand template repair (ssTR) pathway associated primarily with short single-stranded oligodeoxynucleotides used for small sequence changes [62]. Relative to dsDNA donors, long ssDNA induces less cGAS-STING activation and can improve T-cell viability at equivalent donor concentrations [11,53].

Addition of truncated Cas9 target sequences (tCTS) to donor ends provides a strategy to improve non-viral HDR by promoting Cas9 ribonucleoprotein (RNP) binding and donor nuclear co-localisation. This approach increased HDR approximately two- to fourfold across primary cell types and increased the recovery of viable edited cells at reduced donor concentrations [40]. The same principle was subsequently adapted to hybrid single-stranded Cas9 target sequence (ssCTS) templates, in which long ssDNA is flanked by short double-stranded Cas9-binding regions [39].

Compared with fully double-stranded CTS-containing templates, ssCTS increased both knock-in efficiency and edited-cell yield, generating up to sevenfold more knock-in cells under optimised conditions [39]. For a ~2.9 kb anti-BCMA CAR targeted to *TRAC*, ssCTS achieved up to 39% knock-in while maintaining T-cell viability and functional activity, although integration efficiency remained lower than rAAV6 in a matched comparison [39]. The platform was subsequently demonstrated in a GMP-compatible, fully non-viral manufacturing workflow, with further increases in knock-in achievable using HDR enhancers at the expense of live-cell yield [39].

Although tCTS, ssCTS, and Cas9-cleavage sequences (CCS) use related Cas9-assisted donor strategies, they represent distinct donor configurations. tCTS are appended to linear dsDNA donors to promote Cas9-assisted nuclear trafficking [40], whereas ssCTS combine long ssDNA with short double-stranded Cas9-binding regions [39]. By contrast, CCS are incorporated into circular plasmid donors to facilitate generation of a linear HDR substrate, as discussed in Section 3.3 [59]. These terms therefore describe different donor architectures and should not be used interchangeably.

### 3.6. Cas9-VLPs: Electroporation-Free Immune Cell Engineering

A mechanistically distinct approach to CAR T-cell engineering uses engineered virus-like particles to deliver genome editing machinery without electroporation [63]. In the initial Cas9-VLP implementation, Cas9 protein was packaged within HIV-1-based particles, while a lentiviral transfer construct delivered the CAR transgene. In primary human T-cells, this system enabled targeted disruption of *TRAC* or *B2M* together with CAR delivery in a single workflow, generating cells with cytolytic activity against CD19-positive targets [63].

Importantly, this initial Cas9-VLP implementation did not constitute site-specific CAR knock-in. The Cas9 RNP mediated targeted gene disruption, whereas the CAR transgene underwent conventional semi-random lentiviral integration [63]. The platform therefore combined site-specific genome editing with non-site-specific transgene delivery.

More recent work has extended the enveloped delivery vehicle concept to true site-specific CAR integration. A two-component system combining a *TRAC*-targeted Cas9 EDV with an AAV-delivered HDR donor enabled promoterless CAR integration at the *TRAC* locus, including direct generation of site-specifically engineered CAR T-cells in vivo in humanised mouse models [64]. This development demonstrates how transient particle-based nuclease delivery can be coupled with a separate donor platform to achieve targeted CAR integration rather than gene disruption alone.

### 3.7. Off-Target Genotoxicity: Assessment Methods and Clinical Standards

Genotoxicity associated with double-strand break (DSB)-based genome engineering extends beyond conventional off-target indel formation and encompasses a spectrum of unintended genomic alterations. CRISPR-Cas9-induced DSBs can generate large deletions and complex genomic rearrangements that may not be captured by conventional short-range amplicon sequencing [43,65]. Simultaneous DSBs can additionally generate chromosomal translocations, as demonstrated in multiplex-edited T-cells [66]. At the chromosome scale, CRISPR-Cas9 editing can produce abnormalities including aneuploidy, chromosomal truncation, and partial or whole chromosome loss [67,68]. Consequently, safety assessment of site-specific CAR-T engineering requires evaluation of genomic integrity across multiple biological scales rather than relying solely on indel quantification.

Evidence accumulated over the last decade has demonstrated that indel-based assessment alone is insufficient to characterise genome editing-associated risk. CRISPR-Cas9-mediated DSBs can generate large deletions spanning hundreds to thousands of base pairs, as well as complex genomic rearrangements that are not captured by conventional short-range amplicon sequencing [43]. Extending these findings to therapeutically relevant cells, large deletions exceeding 100 bp have been quantified in primary human T-cells following Cas9 editing using an optimised long-range amplicon sequencing workflow, at frequencies markedly higher than those observed with base or prime editors [65]. At the chromosome scale, frequent aneuploidy and chromosomal truncation, including loss of the *TRAC*-bearing chromosome 14, have been demonstrated following Cas9 cleavage in primary human T-cells [68]. Partial or complete chromosome loss has also been observed across multiple target sites and can persist for weeks in culture, including in engineered CAR T-cells [67]. Importantly, modifications to the manufacturing process substantially reduced chromosome loss, indicating that genotoxicity is influenced not only by nuclease specificity but also by cellular processing conditions [67].

Given the diverse nature of these risks, comprehensive safety assessment requires a complementary set of orthogonal methodologies. Genome-wide DSB detection can be achieved using GUIDE-seq in living cells [69] or through biochemical approaches such as CIRCLE-seq and SITE-seq, which identify potential nuclease cleavage substrates prior to cellular validation [70]. Quantitative assessment of editing frequencies at predefined loci is typically performed using rhAmpSeq or targeted deep sequencing [70]; however, these approaches do not adequately detect large structural or chromosomal abnormalities. Methods with broader structural resolution are therefore required: UDiTaS enables simultaneous quantification of indels and structural rearrangements—including large deletions, inversions, and translocations—from a single assay [71], while CAST-Seq was specifically developed to identify and quantify chromosomal aberrations associated with genome editing [72]. Donor integration-site mapping and assessment of unintended donor capture require genome-wide integration profiling such as targeted locus amplification (TLA) [26]. Evaluation of chromosome-scale integrity may therefore require dedicated cytogenetic or genome-wide approaches capable of detecting abnormalities not captured by indel-focused assays [67].

Available clinical and preclinical evidence provides an initial framework for evaluating genomic safety, although important limitations and evidence gaps persist. Preclinical studies of *TRAC*-targeted HDR- and HITI-based CAR knock-in have reported favourable genomic-safety profiles using the assays employed, although the scope and sensitivity of genomic assessment varied between studies [8,26]. In the first-in-human CRISPR-engineered T-cell trial, no off-target editing was detected at predefined high-risk sites and no major safety signals were reported during follow-up. However, low-frequency chromosomal translocations among the multiplex-edited loci were detected, with their frequency declining progressively after infusion [66]. Nevertheless, the study involved a small number of patients and was not designed to fully evaluate rare chromosomal abnormalities or long-term clonal evolution [66].

Taken together, comprehensive safety assessment of DSB-based CAR-T engineering may require a combination of genome-wide off-target cleavage profiling, quantitative indel analysis, chromosomal rearrangement and translocation detection, donor integration-site mapping, and chromosome-scale integrity assessment. While these orthogonal approaches substantially improve risk characterisation, a critical unresolved question remains the long-term in vivo behaviour of edited cell populations harbouring rare or below-detection-threshold genomic alterations, and whether such alterations influence clonal fitness or oncogenic potential during prolonged persistence. Addressing this gap will require longitudinal clinical monitoring together with continued refinement of genome-wide safety-assessment methodologies.

### 3.8. Comparative Analysis (Table 2) and Field Direction

Table 2 compares the principal donor formats used for site-specific CAR integration according to knock-in efficiency, repair pathway, manufacturing considerations, and major advantages and limitations. Reported efficiencies should be interpreted cautiously because they vary with genomic locus, transgene size, donor dose, editing conditions, and post-editing enrichment. Notably, the reported advantage of nanoplasmid-HITI over nanoplasmid-HDR reflects increased CAR T-cell yield within a matched experimental system rather than a general superiority in knock-in efficiency [26].

**Table 2:** Comparative characteristics of donor formats for site-specific CAR integration.

Donor Format	Unselected KI	Pathway	Manufacturing	Main Advantage	Main Limitation
rAAV6	>40% at *TRAC* [8]; 50.7% in matched comparison [39]	HDR	Complex/high cost	High, established KI performance	Limited cargo capacity; GMP manufacturing complexity [44,45]
Linear dsDNA	Variable; improved with tCTS [11,40]	HDR	Simple/low cost	Simple design; no viral packaging limit	Cytotoxicity and innate DNA sensing [11,53]
Conventional plasmid	13–44% [58]	HDR; adaptable to HITI	Simple/low cost	Scalable; flexible cargo	Backbone-associated cytotoxicity [53,57,58]
Nanoplasmid	14–35% HITI [26]; 36–47% HDR [58]	HDR; HITI	Low–moderate	Good balance of KI, viability, and yield	Limited clinical experience; enrichment may be needed [26]
Long ssDNA (ssCTS)	Up to ~39%; 46–62% in GMP-compatible workflow [39]	HDR	Moderate–high	Reduced donor toxicity; efficient non-viral KI	Technically demanding production; lower KI than rAAV6 in matched comparison [39]

Note: CAR = chimeric antigen receptor; HDR = homology-directed repair; HITI = homology-independent targeted integration; KI = knock-in; rAAV6 = recombinant adeno-associated virus serotype 6; ssCTS = single-stranded Cas9 target sequence; tCTS = truncated Cas9 target sequence. KI values are unselected unless otherwise indicated and should not be directly ranked across studies because experimental conditions differ.

Key messages:

- rAAV6-HDR achieves high unselected KI efficiency but carries substantial GMP manufacturing complexity and cost.
- Non-viral donors reduce viral-vector dependence and expand cargo flexibility, but efficiency and cytotoxicity vary by donor format.
- Donor selection therefore reflects a trade-off among KI efficiency, payload capacity, manufacturing feasibility, and cost.

The field is increasingly exploring non-viral alternatives to rAAV6-HDR, driven by manufacturing considerations, cargo flexibility, and the potential to reduce dependence on viral-vector production rather than by a consistent superiority in knock-in efficiency. Emerging transcriptomic evidence suggests that electroporation-based and lentiviral CAR T-cell manufacturing can generate distinct product phenotypes, although the clinical significance of these differences requires prospective validation [73]. rAAV6 is likely to remain an important donor platform because of its established performance and high knock-in efficiency, while non-viral formats may provide advantages in cargo flexibility and manufacturing. Among emerging non-viral strategies, nanoplasmid-HITI combined with pharmacological enrichment and intron-targeted integration coupled to marker-free TCR-negative enrichment illustrate complementary approaches to improving final product purity [26,74]. Broader translation will require independent GMP validation and prospective clinical evaluation.

## 4. Optimal Target Loci for CAR Integration

Where in the genome should the CAR transgene be inserted? The ideal locus would: (i) support high, regulated CAR expression; (ii) avoid disruption of essential genes while functioning as a genomic safe harbour; and (iii) ideally provide additional therapeutic benefits, such as TCR disruption or immune checkpoint knockout. Eight candidate genomic loci relevant to site-specific CAR engineering are summarised in Table 3.

### 4.1. TRAC—T-Cell Receptor Alpha Constant

The *TRAC* gene (chromosome 14q11) is the most established and extensively characterised target for site-specific CAR T-cell engineering [8,17]. CAR integration into the *TRAC* locus simultaneously disrupts the TCR α chain, abolishing surface TCR/CD3 expression, eliminating TCR-mediated alloreactivity, and reducing the risk of graft-versus-host disease (GvHD) in allogeneic settings [8,22,75,76].

Placement of the CAR transgene under endogenous *TRAC* regulatory control provides a major functional rationale for this locus. Compared with conventional lentiviral CAR expression, *TRAC*-targeted integration has been associated with reduced tonic signalling, physiological antigen-dependent CAR internalisation and re-expression, reduced exhaustion, and improved antitumour activity in preclinical models [8]. These effects are attributed to dynamic CAR expression governed by endogenous *TRAC* regulatory elements and have been further supported by studies examining activation-dependent CAR expression and signalling [21]. TCR-deficient T-cells retain effector function, cytokine production, and proliferative capacity [8,22].

The clinical translation of *TRAC*-targeted genome editing is well advanced, although it is important to distinguish two mechanistically distinct approaches. The majority of current clinical programmes employ *TRAC* disruption, using TALENs, CRISPR/Cas9, or other engineered nucleases, to reduce GvHD, while delivering the CAR transgene separately by lentiviral transduction [77]. This paradigm is exemplified by Allogene Therapeutics’ ALLO-501A (cemacabtagene ansegedleucel) [78], CRISPR Therapeutics’ CTX110 [79], and the TALEN- and CRISPR-edited UCART19 platform [75,80]. These programmes support the clinical feasibility of *TRAC* disruption for allogeneic T-cell manufacturing. However, they do not represent site-specific CAR knock-in because the CAR integrates semi-randomly through lentiviral integrase rather than into the *TRAC* locus itself. In contrast, true site-specific CAR integration directs the CAR cassette to the *TRAC* locus, enabling defined genomic placement and, depending on the integration architecture, endogenous regulatory control [8,11,22]. Importantly, *TRAC*-targeted site-specific CAR insertion has now progressed to clinical evaluation. The current clinical maturity of these approaches is discussed in Section 6.

A manufacturing-oriented refinement of *TRAC*-targeted integration involves insertion of the CAR as a synthetic exon within a *TRAC* intron, flanked by engineered splice acceptor and donor sequences [74]. Unlike conventional exonic targeting, NHEJ-mediated indels in cells that fail to undergo HDR do not disrupt the *TRAC* coding sequence, allowing residual TCR-expressing cells to be depleted to enrich successfully integrated TCR-negative cells. This architecture enabled enrichment of CAR^+^ T-cells to >90% purity through TCR/CD3 depletion without additional selection transgenes, drug-resistance genes, or fluorescence-activated cell sorting. The approach was compatible with both Cas9 and Cas12a and was extended to introns within several endogenous surface-receptor genes [74]. Rather than representing a distinct genomic target, intronic integration illustrates how target-site architecture can refine the established *TRAC* paradigm by preserving endogenous regulation while simplifying enrichment of successfully integrated cells.

### 4.2. PDCD1—PD-1 Locus

PD-1 (*PDCD1*; chromosome 2q37) is minimally expressed in resting T-cells and strongly induced following T-cell activation. Targeting CAR or accessory transgenes to the *PDCD1* locus can therefore couple transgene expression to endogenous activation-dependent regulatory programmes while simultaneously disrupting the PD-1 checkpoint. *PDCD1*-targeted integration has been demonstrated in endogenous-locus repurposing studies [38,54], while the functional rationale for simultaneous checkpoint disruption is supported by evidence that cell-intrinsic PD-1 blockade can improve CAR T-cell function in immunosuppressive tumour environments [81].

The rationale for this dual-function strategy derives from the inhibitory role of PD-1 signalling in activated T-cells. Cell-intrinsic PD-1 blockade has been shown to improve CAR T-cell effector function and survival in PD-L1-positive solid tumour models [81]. Integration at *PDCD1* therefore offers two potentially complementary effects: activation-responsive transgene expression under endogenous regulatory control and simultaneous disruption of an inhibitory immune checkpoint.

Importantly, *PDCD1*-targeted CAR integration has progressed beyond preclinical proof-of-concept, with early clinical studies in patients with relapsed or refractory non-Hodgkin lymphoma demonstrating feasibility and encouraging antitumour activity [38,82]. These findings provide early clinical support for *PDCD1* as a site-specific CAR integration locus, although longer-term comparative evidence is required to establish whether activation-coupled CAR expression provides a durable advantage over alternative integration architectures.

This strategy should be distinguished from platforms that combine CAR integration at another locus with independent *PDCD1* disruption. For example, vispacabtagene regedleucel (CB-010) incorporates site-specific CAR insertion at *TRAC* together with PD-1 knockout as a separate editing event [83,84]. Although this architecture also combines CAR engineering with checkpoint disruption, it does not place the CAR transgene under endogenous *PDCD1* regulatory control.

### 4.3. CD7—T-Cell Antigen for T-Cell Malignancies

Engineering CD7-targeting CAR T-cells presents a unique manufacturing challenge in T-cell malignancies, as effector T-cells themselves express CD7, leading to fratricide unless CD7 expression is disrupted prior to CAR introduction [85]. The importance of CD7 disruption is further supported by clinical studies showing that disruption of CD7 expression can prevent fratricide and support CAR T-cell expansion and antitumour activity in T-cell malignancies [86,87].

Direct experimental validation of *CD7* locus-targeted CAR knock-in has been demonstrated using a “2-in-1” strategy combining a single guide RNA targeting *CD7* and an AAV-mediated HDR donor encoding a CD7-specific CAR driven by an exogenous EF1α promoter. This approach simultaneously generated *CD7* knockout and CAR knock-in cells, coupling fratricide prevention with CAR delivery in a single editing step, and was compared with two alternative strategies: random retroviral CAR integration combined with *CD7* knockout, and *TRAC*-targeted CAR knock-in combined with CD7 knockout [23]. All approaches effectively eliminated fratricide and enabled robust T-cell expansion, with comparable in vitro cytotoxicity against CD7-positive tumour targets [23]. Critically, in an in vivo xenograft model of T-cell acute lymphoblastic leukaemia, EF1α-driven CAR expression at the *CD7* locus enhanced tumour rejection relative to random retroviral integration [23], providing preclinical evidence for a potential functional advantage of site-specific integration at this locus.

### 4.4. CD3ζ and CD3ε—TCR Complex Component Loci

Beyond *TRAC*, two additional components of the TCR/CD3 signalling complex have been studied as site-specific CAR integration loci: CD3ζ (chromosome 1q23.3, *CD247*) and CD3ε (chromosome 11q23.3, *CD3E*). The CD3ζ chain is a central signalling component of the TCR/CD3 complex and provides the intracellular activation domain used in conventional CAR architectures [88]. The endogenous *CD247* locus has been exploited for non-viral CRISPR-mediated knock-in of a ζ-domain-truncated CAR inserted in-frame within the *CD247* gene, generating a fusion protein that places CAR expression under endogenous *CD247* regulation while disrupting TCR surface expression [89]. Unlike *TRAC*-targeted integration, *CD247* targeting provides an alternative endogenous locus for CAR insertion and may broaden the applicability of site-specific engineering beyond conventional αβ T-cells. In xenograft models, CD3ζ-integrated CD19-CAR T-cells demonstrated antitumour activity comparable to *TRAC*-targeted and lentivirally engineered CAR T-cells, while proof-of-concept editing in NK cells preserved key effector functions [89]. Notably, *TRAC* integration in γδ T-cells generated unexpected CAR^+^/TCRγδ^+^ double-positive cells, whereas CD3ζ targeting avoided this outcome, supporting its broader applicability across alternative immune effector cell types [89]. An additional advantage is the reduced CAR cassette size, as the endogenous CD3ζ signalling domain eliminates the need to encode this domain within the donor template, thereby reducing HDR payload requirements. Although promising, this strategy remains at the preclinical proof-of-concept stage, and efficient CAR knock-in and in vivo validation in NK cells have yet to be achieved.

Evidence for *CD3E* differs from that for the preceding loci because its use as an integration site has primarily involved miniaturised or TCR-coupled antigen-receptor architectures rather than canonical full-length CARs. The most developed application is knock-in of TCR fusion constructs (TRuCs), chimeric receptors that graft antigen-binding specificity onto the preserved CD3ε chain while retaining the complete endogenous TCR signalling cascade, rather than relying on the synthetic CD3ζ chain and costimulatory domains of conventional CARs. CRISPR-Cas12a-mediated knock-in of an HLA-A2-specific scFv at the *CD3E* locus generated antigen-redirected regulatory T-cells while preserving TCR/CD3 complex surface expression and function [90]. A miniaturised CAR architecture, consisting of the scFv antigen-binding domain fused upstream of the preserved CD3ε sequence without exogenous signalling or costimulatory domains, has also been integrated at the *CD3E* locus and compared directly with a conventional first-generation CAR integrated at the *TRAC* locus. Functional cytotoxicity was demonstrated in primary human T-cells [91]. In the reported *CD3E*-targeted architectures, TCR/CD3 surface expression and endogenous signalling were preserved [90,91]. No canonical full-length CAR has yet been integrated at the *CD3E* locus. Current evidence is limited to TCR-coupled and miniaturised receptor architectures, and the locus should therefore be regarded as an emerging proof-of-concept target.

### 4.5. AAVS1, CCR5, and Extragenic Safe Harbours

The AAVS1 safe harbour (chromosome 19q13, *PPP1R12C* locus) was among the first genomic sites investigated for site-specific transgene integration in T-cells. AAVS1 has been explored as a genomic safe-harbour locus for targeted CAR integration [17]. However, unlike loci such as *TRAC* or *PDCD1*, AAVS1 does not inherently couple CAR integration to disruption of a therapeutically relevant gene or to T-cell-specific endogenous regulation, which may limit its functional advantage as an integration site. It nevertheless remains a useful neutral landing site when locus-specific functional modulation is not required.

*CCR5* (chromosome 3p21) was among the earliest loci investigated for site-specific CAR knock-in using a non-CRISPR nuclease. Early studies demonstrated successful megaTAL-mediated targeted integration at the *CCR5* locus in haematopoietic cells and its subsequent application to anti-HIV CAR T-cell engineering [36,37]. *CCR5* disruption also confers resistance to HIV by inactivating the principal HIV co-receptor, making this locus relevant to HIV-specific CAR T-cell approaches [36,37]. Unlike *TRAC* or *PDCD1*, however, the functional value of *CCR5* as an integration locus is context dependent. Its strongest rationale lies in applications where simultaneous *CCR5* disruption provides an additional therapeutic benefit, particularly HIV-directed cellular therapies [36,37].

An important emerging category is computationally identified extragenic safe harbours, genomic loci selected by genome-wide criteria including chromatin accessibility, transcriptional permissivity, absence of oncogenic neighbourhoods, and stable expression across T-cell differentiation states, rather than by functional gene disruption. One such candidate, GSH6, is located within the non-coding pseudogene *ZNF767P* on chromosome 7 and was identified through multi-criteria computational screening before preclinical validation using CRISPR/Cas9- and rAAV6-mediated CAR knock-in in primary human T-cells [92]. CAR T-cells generated at GSH6 demonstrated sustained CAR expression following repeated antigen stimulation and antitumour activity comparable to *TRAC*-targeted CAR T-cells in preclinical models [92]. These findings illustrate a distinct strategy for locus selection in which genomic sites are chosen for transcriptional stability and genomic context rather than for disruption or repurposing of an endogenous T-cell gene. However, GSH6 remains a preclinical candidate, and its long-term genomic safety and clinical performance require further validation.

The functional characteristics, potential advantages, and limitations of the candidate genomic loci discussed above are summarised in Table 3.

**Table 3:** Comparative functional characteristics of candidate genomic loci for site-specific CAR integration.

Locus (Gene)	Functional Outcome of CAR KI	Key Advantages (+)/Limitations (−)
*TRAC*	TCR KO + endogenous CAR regulation	**+** TCR-KO/CAR-KI in one edit; reduced tonic signalling/exhaustion [8,21]. **+** Intronic KI enables >90% CAR^+^ enrichment [74]. **−** Limited applicability to non-αβ effector cells.
*PDCD1* (PD-1)	PD-1 KO + activation-coupled CAR expression	**+** Checkpoint KO with activation-coupled CAR regulation [38,54]. **+** 86% CR in 21-patient NHL cohort [82]. **−** Low basal CAR expression **−** Separate TCR KO for allogeneic use.
*CD7*	CD7 KO + EF1α-driven CAR KI; prevents fratricide	**+** Single-edit KO/KI [23]. **+** Improved tumour control vs. random RI [23]. **−** Primarily relevant to CD7^+^ malignancies. **−** Exogenous CAR regulation.
CD3ζ (*CD247*)	TCR disruption + CAR expression from CD3ζ promoter	**+** Potential applicability to γδ T and NK cells [89]. **+** Smaller donor payload. **−** Preclinical only.
*CD3E* (CD3ε)	TCR-coupled/miniaturised receptor KI with preserved TCR/CD3 complex	**+** Preserves endogenous TCR signalling [90]. **+** Potential Treg application. **−** Alloreactivity retained. **−** Evidence limited to TCR-coupled/miniCAR architectures [90,91].
AAVS1 *(PPP1R12C)*	Safe-harbour KI + exogenous CAR expression	**+** Established safe harbour [17]. **−** No TCR/checkpoint KO. **−** No additional functional benefit.
*CCR5*	CCR5 KO + CAR KI	**+** HIV resistance [36]. **+** Early site-specific CAR KI proof-of-concept using megaTAL [36,37]. **−** Proof-of-concept; no oncology clinical programme.
GSH6 (*ZNF767P* pseudogene)	Extragenic safe-harbour KI	**+** >80% cleavage; CAR expression comparable to *TRAC* [92]. **+** Durable preclinical activity [92]. **−** No TCR/checkpoint benefit. **−** Preclinical only.

Table footnote: CAR = chimeric antigen receptor; CR = complete remission; KI = knock-in; KO = knockout; NHL = non-Hodgkin lymphoma; NK = natural killer; PD-1 = programmed cell death protein 1; RI = random integration; TCR = T-cell receptor; Treg = regulatory T cell.

Key messages:

- *TRAC* remains the most extensively characterised locus, combining TCR disruption with endogenous CAR regulation.
- Alternative loci offer distinct functional advantages, including checkpoint disruption, fratricide prevention, lineage flexibility, or preservation of endogenous TCR function.
- No single locus is universally optimal; selection depends on CAR architecture, cell type, disease context, and intended therapeutic function.

## 5. Emerging CAR T-Cell Engineering Paradigms and Applications

Most clinically developed CAR T-cell therapies rely on ex vivo manufacturing, in which T-cells are isolated, genetically engineered, expanded under controlled conditions, and reinfused into the patient [6,17]. Site-specific genome engineering is extending this conventional paradigm across autologous and allogeneic CAR T-cell platforms while enabling emerging strategies for direct in vivo T-cell engineering and broader therapeutic applications. Among these developments, direct in vivo reprogramming of endogenous T-cells represents an emerging approach that aims to enable CAR generation within the patient without external cell manipulation. Recent preclinical work has demonstrated the feasibility of site-specific CAR integration in vivo in humanised mouse models [64].

### 5.1. Autologous Versus Allogeneic CAR T-Cell Engineering

Autologous and allogeneic CAR T-cell products impose distinct genome-engineering requirements that directly influence locus selection and editing strategy. Autologous products are generated from patient-derived T cells and do not require disruption of endogenous TCR expression to prevent alloreactivity. In contrast, allogeneic products derived from healthy-donor αβ T cells typically require disruption of donor TCR expression to reduce the risk of GvHD and may require additional strategies to limit host-versus-graft (HvG) rejection [93,94,95]. Although autologous CAR T-cell therapy remains the clinical standard for approved haematological indications, patient-specific manufacturing can be constrained by production time and variable starting-cell quality. Allogeneic products offer standardised, off-the-shelf production but introduce additional immunological and engineering challenges [93,95].

Site-specific CAR integration may be particularly advantageous in the allogeneic setting when the selected locus contributes directly to the required cellular phenotype. *TRAC*-targeted promoterless knock-in is a prominent example, as CAR insertion simultaneously achieves site-specific CAR integration and disruption of endogenous TCR expression while placing CAR expression under *TRAC* regulatory control [8,11]. Thus, a single targeted integration event can combine precise CAR placement with a key requirement of allogeneic αβ T-cell engineering. By contrast, integration at loci such as *AAVS1* or the computationally identified extragenic safe harbour *GSH6* preserves endogenous TCR expression and therefore requires a separate TCR-disruption step for allogeneic applications, as discussed in Section 4 and Table 3.

This dual function makes *TRAC* particularly attractive for allogeneic CAR T-cell engineering because locus selection contributes directly to the generation of the allogeneic product rather than serving solely as a site for CAR insertion. Additional modifications may still be required to limit HvG rejection, including strategies targeting HLA expression or introducing immune-evasive molecules [94,96].

### 5.2. In Vivo Site-Specific CAR T-Cell Engineering

A recent approach to site-specific in vivo CAR T-cell generation used a dual-vector platform combining T-cell-targeted enveloped delivery vehicles (EDVs) carrying CRISPR–Cas9 ribonucleoproteins with an engineered T-cell-tropic rAAV donor encoding a promoterless *TRAC*-targeted CAR cassette [64]. This approach achieved stable CAR expression under endogenous *TRAC* regulatory control in T-cells following systemic administration. In multiple humanised mouse models, in vivo-generated *TRAC*-CAR T-cells produced durable antitumour activity and outperformed dose-matched lentiviral comparators delivered through the same route. The platform also demonstrated minimal detectable editing in isolated human haematopoietic stem cells in vitro [64].

These findings demonstrate the feasibility of site-specific CAR generation in vivo. However, the extent to which the observed effects were attributable specifically to *TRAC*-targeted integration rather than other features of the delivery platform was not examined directly. In addition, the assessment of unintended editing in haematopoietic stem cells was performed in vitro rather than in the tumour-bearing animal models. Further evaluation at clinically relevant doses will be important for assessing cell-type specificity, genomic safety, and translational scalability.

### 5.3. Targeted Delivery Strategies for In Vivo CAR T-Cell Engineering

The cell-type-selective pseudotyping strategy underlying the EDV platform developed through a series of experimental advances. Early Cas9-containing virus-like particles (Cas9-VLPs) pseudotyped with the HIV-1 envelope glycoprotein, which naturally confers CD4^+^ T-cell tropism, selectively delivered CRISPR cargo to CD4^+^ T-cells while leaving co-cultured CD8^+^ T-cells unmodified in mixed primary T-cell populations ex vivo [63]. This demonstrated that natural viral tropism could be exploited to direct genome editing cargo to a defined lymphocyte subset.

Subsequent work extended this concept by replacing natural viral tropism with antibody-directed targeting. EDVs displaying an anti-CD3 single-chain variable fragment (scFv) enabled targeted delivery of Cas9 ribonucleoproteins to T-cells in vivo, while anti-CD3 engagement also activated resting T-cells to support homology-directed repair [97].

This EDV platform was subsequently paired with an engineered AAV-hT7 capsid optimised for primary human T-cell transduction and carrying a promoterless CAR HDR template, enabling site-specific CAR knock-in at the *TRAC* locus in vivo [64]. Together, these advances established a framework for combining programmable T-cell targeting with site-specific genome engineering in vivo [63,64,97].

In contrast to site-specific genomic integration, an alternative approach to in vivo CAR T-cell generation uses non-integrating mRNA delivery. CD5-targeted lipid nanoparticles (LNPs) delivering modified CAR-encoding mRNA generated functional, transient CAR T-cells in vivo, reducing cardiac fibrosis and restoring cardiac function in a mouse model of heart failure [98]. Because the CAR is expressed from mRNA rather than integrated into the genome, this approach provides transient CAR expression without site-specific genomic modification. Although outside the scope of site-specific CAR insertion, this strategy provides a useful comparator for in vivo engineering, particularly where transient rather than long-term CAR expression is desirable.

### 5.4. Translational Challenges of In Vivo CAR T-Cell Generation

Direct in vivo generation of CAR T-cells could simplify manufacturing by eliminating ex vivo cell isolation, genome editing, expansion, and reinfusion, and may reduce the prolonged activation and culture associated with conventional manufacturing [73]. Three challenges currently limit clinical translation: scale, vector immunogenicity, and cell-type specificity.

The first is scale. The substantially larger target-cell population in humans compared with rodent models presents a major challenge when extrapolating delivery and editing efficiency across species. These differences in scale present a substantial challenge when extrapolating delivery and editing efficiency from rodent models to humans. Humanised mouse models provide proof of concept but do not resolve dose-scaling, biodistribution, or editing-efficiency questions. These limitations highlight the importance of evaluation in appropriate large-animal models before human translation, particularly given previous observations that systemic AAV doses tolerated in rodents produced severe toxicity in non-human primates and piglets [99].

A second challenge is vector immunogenicity. Systemic rAAV administration can provoke innate and adaptive anti-capsid immune responses [100], and pre-existing neutralising antibodies against AAV serotypes, present in ~30–70% of adults, reduce transduction efficiency and may augment inflammatory responses [100,101]. The in vivo CAR platform employed an engineered AAV capsid (AAV-hT7) optimised for primary human T-cell transduction [64]; whether it also mitigates pre-existing anti-AAV immunity remains to be established, and prospective screening or alternative-serotype strategies may be required.

The third challenge is cell-type specificity at therapeutic doses. Editing and CAR integration must remain restricted to the intended T-cell population. Unintended integration or CRISPR-induced editing in long-lived progenitors such as haematopoietic stem cells could raise concerns regarding clonal expansion or malignant transformation, informed by previous experience with insertional mutagenesis in retroviral gene therapy [16]. Although EDV delivery is directed by surface-displayed antibody fragments rather than natural viral tropism, cell-type specificity remains to be established at clinically relevant doses [64].

Despite these challenges, in vivo T-cell genomic-engineering programmes are attracting substantial translational and commercial interest [102]. Realising their clinical potential will require rigorous assessment of biodistribution, genomic safety, immunogenicity, and dose scalability.

### 5.5. Site-Specific Genome Engineering for Solid Tumours

Site-specific genome engineering may provide strategies to address several biological barriers that limit CAR T-cell efficacy in solid tumours, including antigen heterogeneity, immunosuppressive signalling, poor tumour infiltration, limited T-cell persistence, hypoxia, and metabolic stress [103]. Although evidence remains predominantly preclinical, recent studies indicate that the functional advantages of targeted integration may extend beyond haematological malignancies.

Limited persistence and progressive exhaustion are major barriers to sustained CAR T-cell activity in solid tumours [103]. The reduction of tonic signalling and exhaustion through endogenous promoter-controlled CAR expression was established at the *TRAC* locus in haematological models [8] and has subsequently been evaluated in solid-tumour settings. Virus-free CRISPR-mediated *TRAC* knock-in of an anti-GD2 CAR promoted a memory-associated phenotype, reduced tonic signalling before antigen exposure, and induced tumour regression in a neuroblastoma xenograft model [104]. Metabolic priming during manufacturing further enhanced memory phenotype retention and persistence of GD2 *TRAC*-CAR T-cells without compromising effector function [105]. However, this latter effect reflects optimisation of the manufacturing environment rather than an intrinsic consequence of site-specific integration.

Site-specific genome engineering may also improve resistance to immunosuppressive signalling within the tumour microenvironment. Non-viral *TRAC*-targeted insertion of a B7-H3 CAR together with c-JUN enhanced activity against low-antigen-density small-cell lung cancer and TGF-β1-secreting *SMARCA4*-deficient tumours [106]. An alternative strategy repurposed tumour-responsive endogenous loci, including *NR4A2* and *RGS16*, for targeted IL-12 or IL-2 insertion in CAR T-cells. This architecture preferentially restricted cytokine expression to intratumoural T-cells and enhanced antitumour activity while limiting peripheral cytokine exposure in preclinical models [107]. Together, these findings illustrate how site-specific engineering can extend beyond CAR placement to incorporate microenvironment-responsive functional programmes designed to counter local immunosuppression.

Antigen heterogeneity and poor tumour infiltration remain additional challenges. B7-H3 is broadly expressed across solid tumours, and *TRAC*-targeted c-JUN/B7-H3 CAR T-cells retained activity at low antigen densities in preclinical models [106]. Evidence for directly improving T-cell trafficking through site-specific engineering is less developed. CRISPR-mediated *CXCL10* knock-in into neuroblastoma cells enhanced CAR T-cell recruitment and antitumour activity in vitro and in humanised mouse models [108]. However, because the tumour cells rather than the CAR T-cells were genetically modified, this provides proof of principle for targeted manipulation of chemokine signalling but not direct evidence for site-specific engineering of CAR T-cell trafficking.

The potential of site-specific integration to improve CAR T-cell fitness under hypoxia and metabolic stress remains underexplored. Intratumoural hypoxia can drive chronic activation of the integrated stress response in CD8^+^ T-cells, promoting mitochondrial oxidative stress and progressive dysfunction [109]. Although this biology identifies potential targets for future engineering, direct evidence that site-specific integration can improve CAR T-cell fitness under hypoxic or metabolically restrictive conditions remains limited.

Collectively, site-specific genome engineering shows promising preclinical potential to improve CAR T-cell performance in solid tumours, particularly through regulation of CAR expression and incorporation of functional programmes that address tumour-associated immunosuppression. Evidence is less developed for improving trafficking and metabolic adaptation. Clinical validation will be required to determine whether these advantages translate into improved patient outcomes.

## 6. Clinical Translation and Manufacturing Readiness

Despite rapid advances in site-specific CAR integration, clinical translation remains substantially less mature than the preclinical literature. Non-viral HDR-mediated integration at the *PDCD1* locus has progressed clinically, with a first-in-human Phase I study reporting a 100% objective response rate and 86% complete response (18/21 patients) in relapsed or refractory non-Hodgkin lymphoma [82]. Site-specific CAR insertion at the *TRAC* locus has also reached clinical evaluation. Vispacabtagene regedleucel (vispa-cel; formerly CB-010) combines site-specific anti-CD19 CAR insertion at *TRAC* with separate *PDCD1* disruption. In the ANTLER Phase I trial, 84 patients with relapsed or refractory B-cell non-Hodgkin lymphoma had been treated; among a 35-patient optimised-profile cohort, the complete response rate was 63%, with 53% progression-free survival at 12 months [83,84]. Other approaches discussed in this review, including HITI, HMEJ, and in vivo site-specific integration, remain predominantly preclinical or proof-of-concept [26,42,64]. Other candidate loci reviewed in Section 4, including *CD7, CD247, CD3E, AAVS1, CCR5,* and *GSH6*, also remain preclinical as site-specific CAR integration targets. This evidence hierarchy illustrates that high editing efficiency alone does not establish translational readiness; manufacturing robustness, scalability, and genomic safety are equally important considerations (Table 4).

Manufacturing readiness does not necessarily parallel biological performance. rAAV6-HDR remains an efficient and extensively characterised donor platform, but its clinical-scale use requires specialised vector production and carries practical cargo constraints after accounting for vector elements and homology arms [44,45]. Non-viral formats, including nanoplasmids and long ssDNA-based donors, may simplify donor production and accommodate larger constructs [39,57]. Research-scale comparisons have reported nanoplasmid donors to be at least fourfold faster to obtain and approximately 20-fold cheaper than dsDNA templates, although these estimates have not been independently validated at equivalent clinical scales [26]. Prospective comparisons of manufacturing cost and production time across donor platforms at equivalent clinical scales are lacking; such estimates should therefore be considered indicative rather than definitive. These potential advantages must also be balanced against template-associated cytotoxicity, variable knock-in efficiency, post-editing enrichment requirements, and the need for reproducible GMP-scale donor production [26,39,58]. These trade-offs become increasingly important as CAR designs incorporate additional functional modules that enlarge the donor cassette.

Locus selection also influences manufacturing complexity. *TRAC* is particularly attractive because CAR integration can simultaneously provide endogenous expression control and disrupt TCR expression in a single editing event [8], potentially simplifying the generation of allogeneic products. A recently described intronic *TRAC* strategy further enables enrichment of CAR-positive cells through TCR/CD3 depletion, potentially simplifying downstream processing [74]. By contrast, extragenic safe-harbour loci preserve endogenous TCR expression and may therefore require an additional TCR-disruption step for allogeneic applications. These considerations illustrate how locus selection can influence not only CAR biology but also the number of engineering and enrichment steps required during manufacturing. The locus-specific biological and technical considerations are discussed in detail in Section 4.

Site-specific editing also adds product-characterisation requirements beyond conventional CAR-T manufacturing. As discussed in the Genomic Safety section, translational development requires confirmation of intended integration and junction fidelity together with assessment of off-target editing, unintended donor integration, chromosomal rearrangements, large structural alterations, and chromosome loss [43,67,69,70,72]. These analyses are relevant to product characterisation and regulatory assessment rather than being solely research-stage considerations. However, standardised approaches and acceptance criteria for rare genomic abnormalities remain incompletely established, complicating comparison across editing platforms.

Manufacturing time and scalability will ultimately influence whether the biological advantages of targeted integration justify the additional process complexity. Conventional autologous CAR-T manufacturing involves patient-specific cell collection, engineering, expansion, product testing, and reinfusion [20]. Site-specific engineering may introduce additional electroporation, enrichment, and genomic-characterisation steps. Conversely, non-viral donor formats may simplify some aspects of template production, while allogeneic platforms could decouple product availability from individual patient manufacturing. In vivo site-specific CAR generation could go further by eliminating ex vivo cell manufacturing altogether, but remains at an early stage and introduces distinct challenges involving cell-type specificity, dose scaling, systemic delivery, and immunogenicity, as discussed in Section 5.4 [64].

Accordingly, integration-strategy selection requires simultaneous consideration of therapeutic indication, target immune-cell type, CAR architecture and cassette size, expression control, autologous versus allogeneic application, genomic safety, and manufacturing feasibility.

Overall, no current site-specific CAR integration strategy simultaneously optimises editing efficiency, payload flexibility, manufacturing simplicity, genomic safety, and clinical maturity. Translation will therefore depend less on maximising any single parameter than on achieving a reproducible balance among these characteristics under GMP-compatible conditions. Continued progress will require scalable manufacturing, rigorous genomic characterisation, and, most importantly, prospective clinical evidence demonstrating that the functional advantages observed in preclinical models translate into meaningful patient benefit.

Key messages:

- Clinical maturity varies substantially across site-specific CAR integration approaches, with most strategies remaining preclinical.
- Manufacturing readiness depends on donor format, GMP compatibility, scalability, payload requirements, and process complexity.
- No current approach simultaneously optimises knock-in efficiency, manufacturing simplicity, scalability, and clinical maturity.

## 7. Conclusions

Site-specific genome engineering has reframed CAR T-cell therapy from a question of transgene delivery to one of transgene design. Integration locus, repair mechanism, donor platform, and expression architecture can influence CAR expression, T-cell phenotype, and therapeutic function. However, no single integration strategy is universally optimal, and potential advantages must be balanced against genomic safety, payload requirements, manufacturing feasibility, and clinical maturity.

Despite substantial preclinical progress, the evidence base remains uneven. Clinical experience is limited to a small number of site-specific approaches, while many alternative loci and integration technologies remain at the proof-of-concept or preclinical stage. Whether improvements in CAR regulation, persistence, exhaustion resistance, and functional consistency translate into superior and durable clinical outcomes remains to be established. Genomic safety also requires further investigation, particularly in light of evidence of chromosome loss and structural abnormalities following nuclease-mediated editing [43,67]. These findings reinforce the need for rigorous assessment of chromosomal integrity as site-specific products advance clinically.

Recent demonstrations of in vivo site-specific CAR integration further extend the potential of targeted engineering beyond conventional ex vivo manufacturing, although clinical translation will require validation of delivery specificity, scalability, immunogenicity, and genomic safety [64]. Future progress will depend on integrating these considerations rather than optimising individual engineering parameters in isolation. Ultimately, the value of site-specific CAR engineering will be determined not by editing efficiency alone, but by whether precise genomic control can be translated into safe, scalable, and clinically meaningful improvements in CAR T-cell therapy.

## Figures and Tables

**Table 1:** Comparative characteristics of HDR-, HITI-, and HMEJ-mediated site-specific CAR integration.

Criterion	HDR	HITI	HMEJ
Knock-in efficiency	Typically 20–50% with rAAV6; variable with non-viral donors [8,29,39].	Higher CAR-positive yield than nanoplasmid HDR in one matched comparison [26].	Up to 70%; 52.4% vs. 42.8% for matched HDR in one study [42].
Post-editing viability	May be reduced by activation and donor-associated toxicity [11,39].	Viable products demonstrated after non-viral editing and enrichment; direct matched viability comparisons remain limited [26].	>80% reported with preserved proliferative capacity [42].
Integration/junction precision	High; sequence-defined homology-directed junctions [8,11].	NHEJ-associated junctional indels may occur [25,26].	Precise targeted integration demonstrated preclinically [42].
Genomic safety considerations	DSB-associated genomic alterations remain possible; donor-related risks depend on donor format [43].	NHEJ-associated junctional indels occur; broader genomic safety evidence remains limited [25,26].	Genomic safety evidence remains predominantly preclinical [42].
CAR expression architecture	Supports promoterless endogenous CAR regulation when appropriately designed, as demonstrated at *TRAC* [8,21].	Current *TRAC*-HITI implementation uses exogenous promoter-driven expression [26].	Compatible with targeted CAR insertion; long-term regulatory behaviour remains less characterised [42].
Cargo flexibility	rAAV6 constrained by packaging capacity; non-viral HDR provides greater flexibility [39,44,45].	Not constrained by viral packaging capacity in non-viral donor implementations [26].	Payloads up to 6.7 kb demonstrated [42].
Functional performance	Strong preclinical evidence for regulated expression and durable function, particularly at *TRAC* [8,21].	Antitumour activity demonstrated; long-term comparative persistence remains limited [26].	Preserved proliferation and antitumour activity demonstrated preclinically; long-term comparative data remain limited [42].

Note: CAR = chimeric antigen receptor; HDR = homology-directed repair; HITI = homology-independent targeted integration; HMEJ = homology-mediated end joining; KI = knock-in; NHEJ = non-homologous end joining; rAAV6 = recombinant adeno-associated virus serotype 6; *TRAC* = T-cell receptor alpha constant. Reported knock-in efficiencies derive from different target loci, CAR constructs, donor formats, and experimental conditions and should not be interpreted as direct cross-study comparisons unless explicitly stated as matched comparisons.

**Table 4:** Clinical Translation Readiness of Site-Specific CAR Integration Approaches.

Site-Specific Integration Approach	Representative Application	Clinical Maturity	Manufacturing Readiness
rAAV6-HDR targeted knock-in	*TRAC* and other targeted loci	Predominantly preclinical; extensive functional evidence [8,22].	Moderate–High; established AAV manufacturing infrastructure, but specialised production and payload constraints remain [44,45].
Non-viral HDR at *PDCD1*	CAR KI + checkpoint disruption	Early clinical; first-in-human Phase I, 100% ORR and 86% CR (*n* = 21) [82].	Moderate; clinical feasibility demonstrated, although process standardisation remains necessary [82].
*TRAC*-targeted CAR insertion + separate *PDCD1* KO (vispa-cel)	Allogeneic TCR-negative anti-CD19 CAR T-cells + PD-1 disruption	Phase I; 84 patients treated; 63% CR and 53% 12-month PFS in the optimised-profile cohort (*n* = 35) [83,84].	Moderate–High; allogeneic clinical manufacturing demonstrated in Phase I [83,84].
Non-viral HDR at *TRAC*	Endogenous CAR regulation + TCR disruption	Advanced preclinical; no published patient outcomes for non-viral *TRAC*-targeted HDR [8,74].	Moderate–High; non-viral delivery and TCR/CD3-depletion enrichment demonstrated [39,74].
HITI-mediated targeted integration	Non-viral CAR insertion at *TRAC*	Preclinical [26].	Moderate; fully non-viral workflow with clinical-scale enrichment demonstrated, but additional processing is required [26].
HMEJ-mediated targeted integration	Targeted integration of larger CAR cargos	Preclinical/proof-of-concept [42].	Early; GMP standardisation and clinical-scale validation remain limited [42].
In vivo site-specific CAR integration	*TRAC*-targeted CAR generation without ex vivo manufacturing	Advanced preclinical; demonstrated in humanised mouse models [64].	Early; avoids ex vivo manufacturing in principle, but human-scale delivery remains unvalidated [64].

Table footnote: CR = complete response; GMP = Good Manufacturing Practice; HDR = homology-directed repair; HITI = homology-independent targeted integration; HMEJ = homology-mediated end joining; KI = knock-in; KO = knockout; ORR = objective response rate; PFS = progression-free survival; TCR = T-cell receptor. Rows represent different levels of the site-specific engineering workflow, including donor platforms, integration mechanisms, locus-specific strategies, and representative clinical products, and are therefore not mutually exclusive. Manufacturing readiness and scalability represent qualitative assessments based on the cited literature rather than formal manufacturing readiness level (MRL) scores, which have not been reported for these platforms.

## Data Availability

No new data were created or analyzed in this study. Data sharing is not applicable to this article.

## Abbreviations

AAV	Adeno-Associated Virus
AAV6	Adeno-Associated Virus Serotype 6
ALLO-647	Anti-CD52 Monoclonal Antibody Used for Lymphodepletion
ARCUS	Proprietary Meganuclease Genome Editing Platform
ASH	American Society of Hematology
BE	Base Editor
*B2M*	Beta-2 Microglobulin
CAR	Chimeric Antigen Receptor
CAR-T	Chimeric Antigen Receptor T-Cell
CAST-seq	Chromosomal Aberration Analysis by Single Targeted Sequencing
CCS	Cas9 Cleavage Sequence
CEMENT	CRISPR EnrichMENT
CR	Complete Response
CRISPR	Clustered Regularly Interspaced Short Palindromic Repeats
CRS	Cytokine Release Syndrome
CTX110	CRISPR Therapeutics Allogeneic Anti-CD19 CAR-T Product
DSB	Double-Strand Break
dsDNA	Double-Stranded DNA
EDV	Enveloped Delivery Vehicle
EGFRt/tEGFR	Truncated Epidermal Growth Factor Receptor
FDA	United States Food and Drug Administration
GFP	Green Fluorescent Protein
GMP	Good Manufacturing Practice
GSH	Genomic Safe Harbour
GSH6	Genomic Safe Harbour 6
GUIDE-seq	Genome-Wide Unbiased Identification of Double-Strand Breaks Enabled by Sequencing
GvHD	Graft-versus-Host Disease
HDR	Homology-Directed Repair
HITI	Homology-Independent Targeted Integration
HMEJ	Homology-Mediated End Joining
HSC	Haematopoietic Stem Cell
ICANS	Immune Effector Cell-Associated Neurotoxicity Syndrome
IND	Investigational New Drug
ITK	Interleukin-2-Inducible T-cell Kinase
KI	Knock-In
KO	Knockout
LBCL	Large B-Cell Lymphoma
LNP	Lipid Nanoparticle
LTR	Long Terminal Repeat
LV	Lentiviral Vector
MHC	Major Histocompatibility Complex
NGS	Next-Generation Sequencing
NHEJ	Non-Homologous End Joining
NK Cell	Natural Killer Cell
ORR	Overall Response Rate
PBMC	Peripheral Blood Mononuclear Cell
PD-1	Programmed Cell Death Protein 1
*PDCD1*	Programmed Cell Death 1 Gene
PFS	Progression-Free Survival
RNP	Ribonucleoprotein
ssTR	Single-Strand Template Repair
ssCTS	Single-Stranded Cas9 Target Sequence
ssDNA	Single-Stranded DNA
TALEN	Transcription Activator-Like Effector Nuclease
TCR	T-Cell Receptor
TLA	Targeted Locus Amplification
tNGFR	Truncated Nerve Growth Factor Receptor
*TRAC*	T-Cell Receptor Alpha Constant
*TRBC*	T-Cell Receptor Beta Constant
VCN	Vector Copy Number
VLP	Virus-Like Particle
WGS	Whole-Genome Sequencing
ZFN	Zinc Finger Nuclease
